# Facial emotion recognition, empathy & psychopathic traits: understanding differential effects of measures

**DOI:** 10.1186/s40359-026-04084-6

**Published:** 2026-01-31

**Authors:** Merve Utanğaç, Karen Lander

**Affiliations:** Division of Psychology, Communication and Human Neuroscience, University of Manchester, Manchester, UK

**Keywords:** Cognitive Empathy, Affective Empathy, Psychopathic Traits, Facial Emotion Recognition, Performance-based Assessment, Self-report Measures

## Abstract

**Background:**

Empathy, encompassing affective and cognitive components, is considered fundamental to understanding psychopathic traits and its relationship with facial emotion recognition. Nonetheless, existing evidence is inconsistent, reflecting variability in measurement approaches and limited use of ecologically valid tasks.

**Methods:**

We examined associations between empathy, psychopathic traits, and facial emotion recognition in 227 participants. Empathy was assessed using both self-report (Questionnaire of Cognitive and Affective Empathy; QCAE) and performance-based measures (Multifaceted Empathy Test; MET). Psychopathic traits were measured with the Psychopathic Personality Traits Scale–Revised (PPTS-R). Facial emotion recognition was tested using dynamic stimuli across ten emotions.

**Results:**

Affective empathy was negatively associated with psychopathic traits across both QCAE and MET. Cognitive empathy showed divergent patterns: self-reported cognitive empathy correlated negatively with psychopathic traits, whereas performance-based cognitive empathy did not. Facial emotion recognition was positively associated only with performance-based cognitive empathy, particularly for embarrassment. No significant associations emerged between psychopathic traits and facial emotion recognition.

**Conclusion:**

These findings demonstrate the importance of incorporating diverse assessment tools to investigate empathy, alongside self-report measures. The study further contributes to the existing literature on the relationships among empathy, psychopathic traits, and facial emotion recognition.

## Introduction

Empathy plays a fundamental role in comprehending social dynamics and forming social relationships, as it requires the understanding and sharing of others’ emotional states [1, 2]. There is broad heterogeneity in how empathy is conceptualized, with Cuff et al. [3] identifying 43 distinct definitions in their review. However, more recent definitions conceptualize empathy as an outcome of interplaying affective and cognitive dimensions [3–7]. These processes are termed ‘affective empathy’, requiring the experience of others’ emotions and ‘cognitive empathy’, which entails the capacity to comprehend others’ feelings [4, 8].

Such differentiation is particularly relevant when examining empathy in clinical populations. For example, individuals with autism spectrum disorder often show intact affective empathy but reduced cognitive empathy [9], whereas those with psychopathy are typically characterized by deficits in affective empathy [10]. However, these patterns are not consistent: Larsen et al. [11] found no consistent or replicable evidence of empathy deficits across psychopathy samples, underscoring the complexity of empathic functioning and the need for further research.

Despite increasing consensus regarding the multidimensional nature of empathy, substantial variability in its conceptualization and measurement remains a significant challenge. Commonly used self-report scales, such as the Empathy Quotient [12] and the Interpersonal Reactivity Index [13] differ in their theoretical bases and in whether they clearly separate affective and cognitive aspects. Such variability complicates cross-study comparisons and contributes to inconsistent findings. To address this, the Questionnaire of Cognitive and Affective Empathy (QCAE [14]) was developed to explicitly differentiate between affective and cognitive components, aligning with the widely accepted view of empathy as a multidimensional construct.

Recent meta-analyses have also emphasized the importance of distinguishing psychopathy from antisocial behavior as the two conditions show partially distinct empathy profiles [15]. Antisocial behavior refers to a broad range of norm-violating actions such as aggression and rule-breaking that disrupt social order [16]. Although often overlapping, it primarily reflects overt behavioral deviance rather than the affective and interpersonal deficits central to psychopathic traits [17]. Psychopathy refers to a cluster of enduring personality traits encompassing affective, cognitive, interpersonal, and egocentric dimensions, characterized by shallow affect, reduced empathy, manipulativeness, and self-centeredness [18]. While individuals with these traits may engage in antisocial acts or crime, such behaviors were not considered essential for its identification [10]. Psychopathy is more strongly associated with deficits in affective empathy, whereas antisocial behavior is linked to greater impairments in cognitive empathy [15]. Importantly, affective empathy deficits in antisocial individuals often emerge only when psychopathic features are also present [19, 20]. To address these distinctions, the present study employed the Psychopathic Personality Traits Scale–Revised (PPTS-R [18]) to examine the various factors within psychopathy, rather than relying on a single total score or classifying individuals into distinct groups. This measure, which excludes overt criminal behaviors, focuses on the core affective and interpersonal traits of psychopathy.

Although a lack of affective empathy is central across most psychopathy models [21], the relationship with cognitive empathy remains unclear [22]. Some studies report negative associations [23], while others find no significant deficiencies [24], and some even suggest superiority in cognitive empathy tasks [25, 26]. Such variability may reflect differences in sample type (forensic vs. community), measurement approaches but also the fact that, as outlined earlier, both empathy and psychopathy consist of multiple interacting components, including the interaction between affective and cognitive empathy [27, 28]. For instance, when empathy is assessed using the Multifaceted Empathy Test (MET [29]), a computer-based task that uses photographic stimuli to measure both cognitive and emotional empathy, studies have shown that psychopathic traits are negatively correlated with emotional empathy, whereas cognitive empathy tends to show no association [18, 30].

Inconsistencies across studies may also arise from differences in how psychopathic traits are operationalized across instruments. Although many widely used measures adopt a broadly similar trait-based framework, they differ in the extent to which they incorporate antisocial or behavioral criteria. For instance, the Psychopathy Checklist–Revised (PCL-R [31, 32]), includes a substantial behavioral component, whereas more recent trait-focused instruments (e.g., PPTS-R [18]) intentionally exclude criminal items to isolate affective–interpersonal features. Such structural differences have been noted as potentially influencing associations with external constructs [17, 33, 34]. In parallel, studies employing self-report empathy questionnaires such as the QCAE have found reduced affective empathy in psychopathy, though results for cognitive empathy remain mixed [24, 35, 36]. This suggests that inconsistencies in the literature may arise not only from variation in empathy assessment, but also from differences in the psychopathy measures employed.

The present study extends previous research by integrating both self-reported (QCAE) and performance-based (MET) measures of empathy to examine how self-perceived and performance-based empathy relate to psychopathic traits assessed with a non-criminal instrument (PPTS-R). These construct-validity limitations in both psychopathy and empathy measures may also help explain the inconsistent associations reported with facial emotion recognition, which is considered crucial for understanding the etiology of psychopathy [37].

### The role of facial emotion recognition

Comprehending facial expressions is a crucial function for detecting social cues and reinforcing socially acceptable conduct. This ability is argued to be closely linked to empathy, thus researchers have explored how individual traits like empathy might influence how people perceive and respond to facial expressions [38]. Research on its association with empathy has produced mixed results: affective empathy shows weak or inconsistent links with recognition accuracy [39–41]. In contrast, cognitive empathy, which goes beyond the interpretation of emotional expressions to involve perspective-taking and a deeper understanding of others’ mental states, has been found to facilitate facial emotion processing [42]. Recent meta-analytic evidence across 38 studies (*N* = 13,082) further confirms a small but positive association between cognitive empathy and facial emotion recognition [43].

In psychopathy, difficulties interpreting others’ emotional distress have been proposed, particularly for negative emotions such as fear and sadness [26–28]. These deficits are most consistently associated with callous–unemotional and antagonistic traits, reflecting reduced sensitivity to others’ suffering [44–46]. Neurophysiological evidence also links callousness to reduced affective engagement during emotion processing [47]. Consistent with this, several studies and meta-analyses report impairments in recognizing these emotions, but relatively intact recognition of happiness, anger, and disgust [39, 41]. Other accounts suggest broader, modality-general impairments, possibly linked to attentional deficits rather than emotion-specific problems [37, 48]. Supporting this, psychopathic offenders show reduced spontaneous activation in empathy-related brain regions when viewing emotions, yet comparable activation to controls when explicitly instructed to empathize [49].

Despite these findings, results remain inconsistent. Some studies have reported no deficits in facial emotion recognition among individuals with psychopathy [50] while others have even found enhanced recognition of certain affective expressions [48]. Trait-specific effects may explain these discrepancies, with antisocial behavior linked to poorer recognition and callous affect was positively associated with identifying sadness expressions, potentially reflecting strategic sensitivity to others’ vulnerability rather than genuine empathic concern [51]. Together, this suggests that emotion recognition in psychopathy is not uniformly impaired but might depend on specific trait profiles and motivational contexts [37, 52, 53].

### The current study

Taken together, the mixed findings regarding the relationship between empathy, psychopathic traits, and facial emotion recognition may stem from two main factors.

*First*,* measurement variability.* Within empathy research, commonly used instruments differ substantially in how they define and assess the construct [54]. Likewise, psychopathy models vary in whether antisocial behaviors and impulsivity are treated as core components or secondary correlates [15]. Such differences in conceptualization and measurement contribute to disparate associations across studies.

*Second*,* methodological limitations.* Many studies rely heavily on self-report measures of empathy, which show limited reliability and ecological validity compared with performance-based assessments [15]. For instance, recent meta-analytical research highlighted concerns about self-report scales’ limited ability to measure cognitive empathy, explaining only 1% of the variance in behavioral assessments [55]. In facial emotion recognition tasks, further variability arises due to differences in stimulus intensity and the common use of static or morphed faces rather than dynamic, naturalistic expressions [56, 57]. Yet empirical evidence suggests that facial motion can provide additional information beyond static cues and may affect recognition performance, particularly when task demands are higher [58].

The present study addresses these two challenges (measurement variability, and methodological limitations) to provide a more precise examination of the relationships among empathy, psychopathic traits, and facial emotion recognition. To minimize measurement variability, we employed the Questionnaire of Cognitive and Affective Empathy (QCAE [14]) to clearly define and measure cognitive and affective empathy within a single, explicitly component-based framework, thereby addressing inconsistencies in how these components are defined across studies. For example, instruments often treated as indexing cognitive empathy, such as the Hogan Empathy Scale and the Perspective Taking subscale of the Interpersonal Reactivity Index, have been critiqued as capturing broad perspective taking rather than cognitive empathy as emotion understanding, which can limit cross-study comparability [54]. This clarification is particularly relevant given that empirical inconsistencies in the psychopathy literature have most often concerned cognitive empathy, which is sometimes conflated with Theory of Mind [15, 59, 60]. We also used the Psychopathic Personality Traits Scale–Revised (PPTS-R [18]) to capture core psychopathic traits within a non-clinical community sample while avoiding direct confounding with antisocial behavior. This distinction is critical, as individuals who present both psychopathic traits and antisocial tendencies represent a distinct subgroup with potentially different emotional processing profiles [15, 53]. Together, these measures align component-level empathy estimates with facet-level psychopathic traits, improving cross-study comparability in a literature where constructs are often operationalized inconsistently.

To enhance ecological validity, dynamic facial expressions varying in intensity across ten discrete emotions were used in the recognition task. Moreover, the Multifaceted Empathy Task (MET [29]) was incorporated alongside the QCAE to capture cognitive empathy through a performance-based behavioral measure and rather than relying solely on self-report instruments. The MET also captures affective empathy, offering a more ecologically valid alternative to traditional questionnaire-based approaches. This allows for a comparison of how self-report and performance-based assessments of cognitive empathy, alongside a contextualized assessment of affective empathy, differentially relate to the association between psychopathic traits and emotion recognition.

Given the heterogeneity and inconsistencies in prior research, the present study adopts an exploratory approach to examine: (a) the correlation between empathy and psychopathic traits, considering both global and facet-level dimensions; (b) how the relationships between individual components of empathy and distinct emotional face recognition abilities; (c) the associations between global and specific psychopathic trait scores and diverse facial emotion recognition outcomes; and (d) potential differences between performance-based and self-report measures of empathy in their associations with psychopathic traits and facial emotion recognition.

## Method

### Design and participants

A within-participants design was used with all participants completing empathy and psychopathic traits questionnaires, as well as an emotion recognition task. A priori power analysis was conducted using G*Power 3.1.9 [61] based on interaction between intensity and accuracy of facial emotion recognition task (the most complex element of the study). To detect a small effect size at a set power of 0.95 and a significance level (alpha) of 0.05, at least 163 participants were required [62]. Given this, the final sample of 230 participants was considered sufficient to ensure representativeness. A total of 230 participants were recruited through two separate platforms: Sona, which is exclusively accessible to undergraduate students enrolled at the University of Manchester, and Prolific (www.prolific.com; all participants were located in the UK), a platform open to individuals who participated in exchange for financial compensation. Participants were recruited from a non-clinical community sample comprising both university students and members of the general population. All data were collected online.

Participants were required to be aged between 18 and 60 years and to use corrective lenses if necessary. Individuals who self-reported a current or past diagnosis of autism spectrum condition, depressive disorder, or anxiety disorder were excluded, as these conditions are known to be strongly related to empathy and/or facial emotion-recognition performance [9, 63–65]. No additional psychiatric or neurological screening was administered. Moreover, participants were instructed to use computers, excluding tablets or phones, and specific browsers (e.g., Google Chrome or Firefox) to enhance data collection quality. Three participants were excluded from the analyses for providing inaccurate responses to the attention check questions. The final sample comprised 227 participants aged between 18 and 60 (*Mage* = 36.61, *SDage* = 12.88). Among these, 117 participants were male (*Mage* = 38.40, *SDage* = 11.48) and 110 participants were female (*Mage* = 34.71, *SDage* = 14.02). Ethical approval was obtained from the University of Manchester’s Division of Psychology, Communication, and Human Neuroscience panel (Ref: 2023–16160-27122). Informed consent was obtained from each participant, and no personally identifiable information was requested.

### Materials

#### Questionnaire of Cognitive and Affective Empathy (QCAE)

The QCAE [14] is a 31-item self-report measure that distinguishes between affective and cognitive components of empathy. Using a 4-point Likert scale, respondents indicated their level of agreement, ranging from “strongly disagree” (scored as 1) to “strongly agree” (scored as 4) [14]. An attention check question was integrated into the questionnaire, prompting participants to select the specified option. The final scores were computed by summing up the total score for affective and cognitive empathy, with higher scores indicating higher levels of empathy. Specifically, the highest possible score for affective empathy was 48, while it was 76 for cognitive empathy.

This assessment yielded scores for affective empathy, which was measured by three subscales. The first subcomponent, emotional contagion (4 items), includes items related to mirroring others’ emotional states in a self-oriented manner when observing their affective states. Proximal responsivity (4 items) measures emotional responsiveness to the affective situations of close social contacts (e.g., friends). Finally, peripheral responsivity (4 items, with three items reverse-scored), assesses how individuals respond to affective situations that arise in a detached context. To assess affective empathy, a sample item was, “People I am with have a strong influence on my mood”. Cognitive empathy encompassed two subscales: perspective taking (10 items), involving the adoption of others’ viewpoints, and online simulation (9 items; one item reverse-scored), which involves a deliberate effort to understand others’ emotional states by imagining their feelings. An example item used to evaluate cognitive empathy was, “I sometimes find it difficult to see things from the ‘other guy’s’ point of view”.

Reniers et al. [14] reported verified convergent and construct validity. In addition, internal consistency exhibits an acceptable fit for affective empathy subscales; *emotional contagion*, α = 0.72; *peripheral responsivity*, α = 0.65; *proximal responsivity*, α = 0.70 and a good fit for cognitive empathy subscales; *perspective taking*, α = 0.85; *online simulation*, α = 0.83. In the present experiment, Cronbach’s α for subscales calculated as follows: *emotional contagion*, α = 0.67; *peripheral responsivity*, α = 0.58; *proximal responsivity*, α = 0.72; *perspective taking*, α = 0.88; *online simulation*, α = 0.86. The QCAE demonstrated strong reliability for the total scale and the cognitive empathy component. The relatively lower values observed for some affective subscales likely reflect their small number of items (four each) and are consistent with prior validation work.

#### Multifaceted empathy task

MET-core-2 [29] is a computer-assisted test that assesses cognitive and affective aspects of empathy. It was utilized to measure various dimensions of empathy, employing online performance-based components that differentiate it from self-report empathy tests like the QCAE. The task primarily focused on extracting information from the individuals’ facial expressions and body language captured in the images, as highlighted by Dziobek et al. [29] Moreover, MET-core-2 employs photorealistic complex emotions that are context-dependent to enhance ecological validity [29]. It consists of 40 photographs depicting individuals in emotionally charged situations, divided into positive (20 items, e.g., satisfied, cheerful, victorious) and negative (20 items, e.g., terrified, hopeless, pained) scenarios. The difficulty of the items, as well as the ages and genders of the individuals in the photographs, was balanced across both the positive and negative stimuli [29]. The size of these images varied for each photograph from a minimum of 334 × 500 pixels to a maximum of 750 × 500 pixels. Initially, participants were asked to infer the mental states of the individuals shown in the photographs and subsequently select the correct emotional state descriptor among four options, constituting the cognitive empathy condition. Following this, participants were instructed to rate the extent to which they empathized with the individual in the same picture, corresponding to the affective empathy condition, using a Likert scale ranging from 0 = not at all to 9 = very much.

The number of correct identifications was counted to give a score for cognitive empathy and the mean rating was computed for affective empathy. The maximum score that could be obtained according to this calculation was 9 for affective empathy and 40 (40 trials) for cognitive empathy. Dziobek et al. [29] conducted a factor-based greatest lower bound (glb) analysis to account for variations in response accuracy among items within the test. They obtained a reliable estimate (glb = 0.75) for the whole scale and reported that Cronbach’s alpha estimate may be excessively conservative. In the present study, only Cronbach’s α was conducted and it was excellent for affective empathy (α = 0.97), and acceptable for cognitive empathy (α = 0.60).

#### Psychopathic Personality Traits Scale– Revised (PPTS-R)

PPTS-R [18] is a self-report psychopathy trait scale used to assess participants’ psychopathic personality traits within a non-forensic population. This measure is an updated version of the initial PPTS proposed by Boduszek et al. [66], designed to assess psychopathic traits across both forensic and non-forensic groups. The scale comprises four subscales: affective responsiveness (AR), cognitive responsiveness (CR), interpersonal manipulation (IPM), and egocentricity (EGO). The AR subscale is utilised to evaluate an individual’s capacity for affective empathy and emotional depth. The CR subscale measures one’s ability to comprehend and represent the emotional states of others and to establish cognitive emotional connections with them. The subscale of IPM is employed to assess traits like superficial charm, grandiosity, and deceitfulness. Lastly, the EGO subscale pertains to the inclination and prioritization of an individual’s own interests and beliefs. Each item was designed to measure knowledge/skills or attitudes/beliefs rather than behaviors [18].

Each subscale consists of 7 items, with responses rated on a 5-point Likert scale ranging from “Strongly disagree” (scored as 0) to “Strongly agree” (scored as 4). Two items are scored in reverse in the assessment of cognitive responsiveness. The scores of the four subscales are aggregated, with elevated scores indicating heightened levels within each respective dimension. The minimum attainable overall score is 0, with a maximum possible score of 112. The internal reliability of the PPTS-R was assessed using both composite reliability and Cronbach’s alpha, both of which indicated excellent internal consistency across all subscales [18]. For the general population, the published internal reliability scores were as follows: *affective responsiveness*, α = 0.95; *cognitive responsiveness*, α = 0.86; *interpersonal manipulation*, α = 0.90; *egocentricity*, α = 0.89. In the present study, Cronbach’s *α* for the four subscales were *affective responsiveness*, α = 0.85; *cognitive responsiveness*, α = 0.67; *interpersonal manipulation*, α = 0.85; *egocentricity*, α = 0.76. The difference in scores may be attributed to the difference in sample sizes [67]. The comparatively lower reliability of the cognitive responsiveness subscale (α = 0.67) should be considered when interpreting findings related to this dimension.

#### Facial emotion recognition task

The facial emotion recognition task involved 160 dynamic facial expression videos selected from the Amsterdam Dynamic Facial Expression Set (ADFES [68]). The videos consisted of recorded emotional expressions recorded by each model (4 male & 4 female). The expressions included neutral and nine emotions: the six ‘basic’ emotions (anger, disgust, fear, joy, sadness, and surprise), as well as three complex emotions (contempt, pride, and embarrassment), each presented at two intensity levels (low & high; apart from neutral) [68]. The videos were divided into two blocks, ensuring that different intensities of emotions were not presented within the same block. The sequence of videos was randomized within each block for each participant, and a break option was provided between blocks. Each video began with a fixation cross displayed for 500 milliseconds and then the expression sequence was shown for 1000 milliseconds, capturing the entire duration of the facial expression from neutral to its peak. The task was displayed at a resolution of 1024 × 768 pixels. After each video, a response screen appeared, presenting a grid of all nine emotions in two columns and five rows, arranged in alphabetical order. Accuracy was scored on each trial as correct (1) or incorrect (0). Total and emotion-specific accuracy scores were calculated as the sum of correct responses. The FER task showed good internal consistency overall (α = 0.90), with good reliability in both the low-intensity (α = 0.80) and high-intensity (α = 0.81) blocks.

### Procedure

The study was conducted on the data collection platform Gorilla (www.gorilla.sc [69]), which facilitated the generation of all questionnaires and tasks. At the start of the experiment, participants were presented with the Participant Information Sheet and the Consent Form. They were given the flexibility to complete the study at their own pace, with an expected duration of approximately 30 min. Upon starting the study, participants were asked to provide their age and gender. Subsequently, they completed the Questionnaire of Cognitive and Affective Empathy (QCAE [14]) and Psychopathic Personality Traits Scale – Revised (PPTS-R [18]). The order of these assessments was randomized among participants using a standard Latin Square design, implemented through the order node in Gorilla.

Following the completion of the QCAE [14] and PPTS-R [18], participants completed the Multifaceted Empathy Task (MET [29]) and Facial emotion recognition Task. Participants had the opportunity to practice the MET prior to its administration. During the practice stage, participants were presented with both cognitive and affective empathy conditions once. This served to familiarize them with the MET procedure and ensure their full comprehension of the requirements. The order of the MET and Facial emotion recognition tasks was randomized among participants using a standard Latin Square design. Furthermore, a break was provided between these two tasks, the duration of which was determined by participants to manage potential participant fatigue and ensure data quality and reproducibility [70]. Upon completing the final stage of the study, participants underwent a debriefing process. The median completion time for the experiment was approximately 27 min.

### Statistical analysis

Data analysis was carried out in R version 4.3.1 [71]. The analytical plan followed a planned sequence beginning with data screening, descriptive statistics, and assumption checks, followed by analyses mapped to each research question. These included (i) independent-samples t tests for gender differences, (ii) Pearson correlation analyses addressing Research Questions a–d with Benjamini–Hochberg correction, (iii) targeted regression follow-ups for outcomes showing significant bivariate associations with FER, and (iv) emotion × intensity effects in FER accuracy examined using a two-way repeated-measures ANOVA with Bonferroni-adjusted post hoc tests. For the regression follow-up, MET cognitive empathy (dependent variable) was modeled using age and overall FER accuracy as predictors; a second model replaced overall FER with the emotion-specific accuracy scores that showed significant correlations (embarrassment and pride), alongside age. Primary predictors were specified a priori based on the theoretical framework; AIC-based stepwise procedures were used only as an exploratory sensitivity check to evaluate whether additional variables improved model fit [72].

Normality and outlier diagnostics (reported for descriptive purposes) indicated no extreme outliers (3 × IQR) and approximately normal distributions (Shapiro–Wilk: all ps > 0.05). Prior to regression analyses, linearity, homoscedasticity, and residual normality were evaluated via standard diagnostic plots. Multicollinearity was assessed via VIFs (all < 5) [73], and independence of residuals via Durbin–Watson tests (all ps > 0.05) [74]. No influential points were detected via leverage diagnostics. To address potential item overlap between self-report measures, a confirmatory factor analysis (CFA) was conducted; full details and model outputs are provided in Appendix 2.

## Results

### Sample characteristics and preliminary analyses

Independent samples t-tests were conducted to examine gender differences across all study variables, including empathy (QCAE, MET), psychopathic traits (PPTS-R subscales and total score), and facial emotion recognition (FER). Full descriptive statistics and gender-based comparisons are presented in Table 1. Participants were aged 18–60 years (M = 36.62, SD = 12.88); men were older than women on average (men: M = 38.40, SD = 11.48; women: M = 34.72, SD = 14.03). Women scored significantly higher than men on QCAE affective empathy, *t*(225) = − 6.37, *p* <.001, and QCAE cognitive empathy, *t*(225) = − 3.18, *p* =.001. No significant gender differences were observed for MET affective or cognitive empathy (both *ps* > 0.05).For psychopathic traits, men scored higher than women on the PPTS-R total score, *t*(225) = 2.45, *p* =.014. Examination of subscales showed that men scored significantly higher on affective responsiveness, *t*(210.92) = 3.76, *p* <.001, and cognitive responsiveness, *t*(224.54) = 2.74, *p* =.007. No significant gender differences were found for EGO or IPM (both *ps* > 0.25).Facial emotion recognition accuracy (FER - Total) did not differ between men and women, *t*(225) = − 1.30, *p* =.192.

**Table 1:** Descriptive statistics and gender differences for all variables

Measure	Total M (SD) [Min–Max]	Men M (SD) [Min–Max]	Women M (SD) [Min–Max]	t	df	*p*
QCAE – Affective Empathy	33.59 (4.91) [17–48]	31.73 (4.30) [17–42]	35.56 (4.76) [22–48]	−6.37	225	< 0.001
QCAE – Cognitive Empathy	58.05 (7.21) [35–76]	56.60 (7.23) [35–74]	59.59 (6.89) [43–76]	−3.18	225	0.001
MET – Affective Empathy	5.85 (1.45) [1.02–8.57]	5.94 (1.50) [1.02–8.57]	5.76 (1.40) [1.73–8.47]	0.93	225	0.350
MET – Cognitive Empathy	27.49 (4.05) [11–37]	27.44 (3.78) [17–37]	27.55 (4.33) [11–36]	−0.18	225	0.851
PPTS-R – Egocentricity	15.80 (4.15) [7–33]	16.10 (4.44) [7–33]	15.48 (3.81) [7–25]	1.13	223.19	0.259
PPTS-R – Affective Responsiveness	12.02 (4.16) [7–28]	12.99 (4.62) [7–28]	10.99 (3.33) [7–21]	3.76	210.92	< 0.001
PPTS-R – Interpersonal Manipulation	16.56 (4.91) [7–32]	16.71 (4.57) [7–28]	16.40 (5.27) [7–32]	0.47	216.08	0.638
PPTS-R – Cognitive Responsiveness	14.24 (3.51) [7–23]	14.85 (3.63) [7–23]	13.59 (3.26) [7–23]	2.74	224.54	.007
PPTS-R – Total Score	58.62 (12.96) [29–98]	60.65 (13.66) [29–98]	56.46 (11.85) [30–81]	2.45	225	0.014
FER – Total Accuracy	106.18 (13.74) [62–136]	105.03 (14.43) [62–135]	107.41 (12.93) [73–136]	−1.30	225	0.192

*QCAE* Questionnaire of Cognitive and Affective Empathy, *AE* Affective Empathy, *CE* Cognitive Empathy, *MET* Multifaceted Empathy Test, *PPTS-R* Psychopathic Personality Traits Scale – Revised, *FER* Facial Emotion Recognition

Age showed negative correlations with QCAE affective and cognitive empathy and positive correlations with MET affective and cognitive empathy (see Table 2). However, it was not significantly correlated with FER total accuracy (*r* =.04, *p* =.588) or PPTS-R total (*r* = −.08, *p* =.235).

**Table 2:** Correlations among QCAE, MET, PPTS-R subscales, and age (Benjamini–Hochberg corrected)

Measure			PPTS-*R* subscales			
	Subscale	Age	AR	CR	IPM	EGO
QCAE	Affective Empathy	− 0.16*	− 0.55***	− 0.43***	− 0.10	− 0.23**
	Cognitive Empathy	− 0.18**	− 0.48***	− 0.75***	− 0.10	− 0.44***
MET	Affective Empathy	0.16*	− 0.40***	− 0.18*	− 0.24***	− 0.24***
	Cognitive Empathy	0.42***	− 0.08	0.04	− 0.09	− 0.11

*QCAE* Questionnaire of Cognitive and Affective Empathy, *MET* Multifaceted Empathy Test, *AR* Affective Responsiveness, *CR* Cognitive Responsiveness, *IPM* Interpersonal Manipulation, *EGO* Egocentricity. * *p* <.05. ** *p* <.01. *** *p* <.001

### Empathy and psychopathic traits

Pearson correlations among empathy measures (QCAE, MET), PPTS-R subscales, and age are presented in Table 2. QCAE affective empathy was negatively correlated with psychopathic traits (PPTS-R total), *r* = −.40, *p* <.001, as was QCAE cognitive empathy, *r* = −.54, *p* <.001. Both components were also negatively associated with AR, CR and EGO, but not IPM (See Table 2). Performance-based affective empathy (MET affective empathy) was also negatively associated with psychopathic traits (Table 2), whereas MET cognitive empathy showed no significant association with psychopathic traits.

Given significant associations of age and gender with several key variables, partial correlations controlling for these variables were computed. The pattern of results remained unchanged: QCAE affective empathy (partial *r* = −.39, *p* <.001), QCAE cognitive empathy (partial *r* = −.55, *p* <.001), and MET affective empathy (partial *r* = −.35, *p* <.001) remained significantly negatively associated with PPTS-R total, whereas MET cognitive empathy remained non-significant (partial *r* = −.04, *p* =.51).

### Facial emotion recognition (FER)

#### FER accuracy by emotion and intensity

A two-way repeated-measures ANOVA tested effects of emotion and intensity on recognition accuracy. Main effects were significant for emotion, *F*(6.37, 1472.2) = 192.63, *p* <.001, ηG² = 0.33, and intensity, *F*(1, 231) = 1663.94, *p* <.001, ηG² = 0.17. The emotion × intensity interaction was also significant, *F*(7.15, 1652.6) = 57.08, *p* <.001, ηG² = 0.05.

**Fig. 1:**
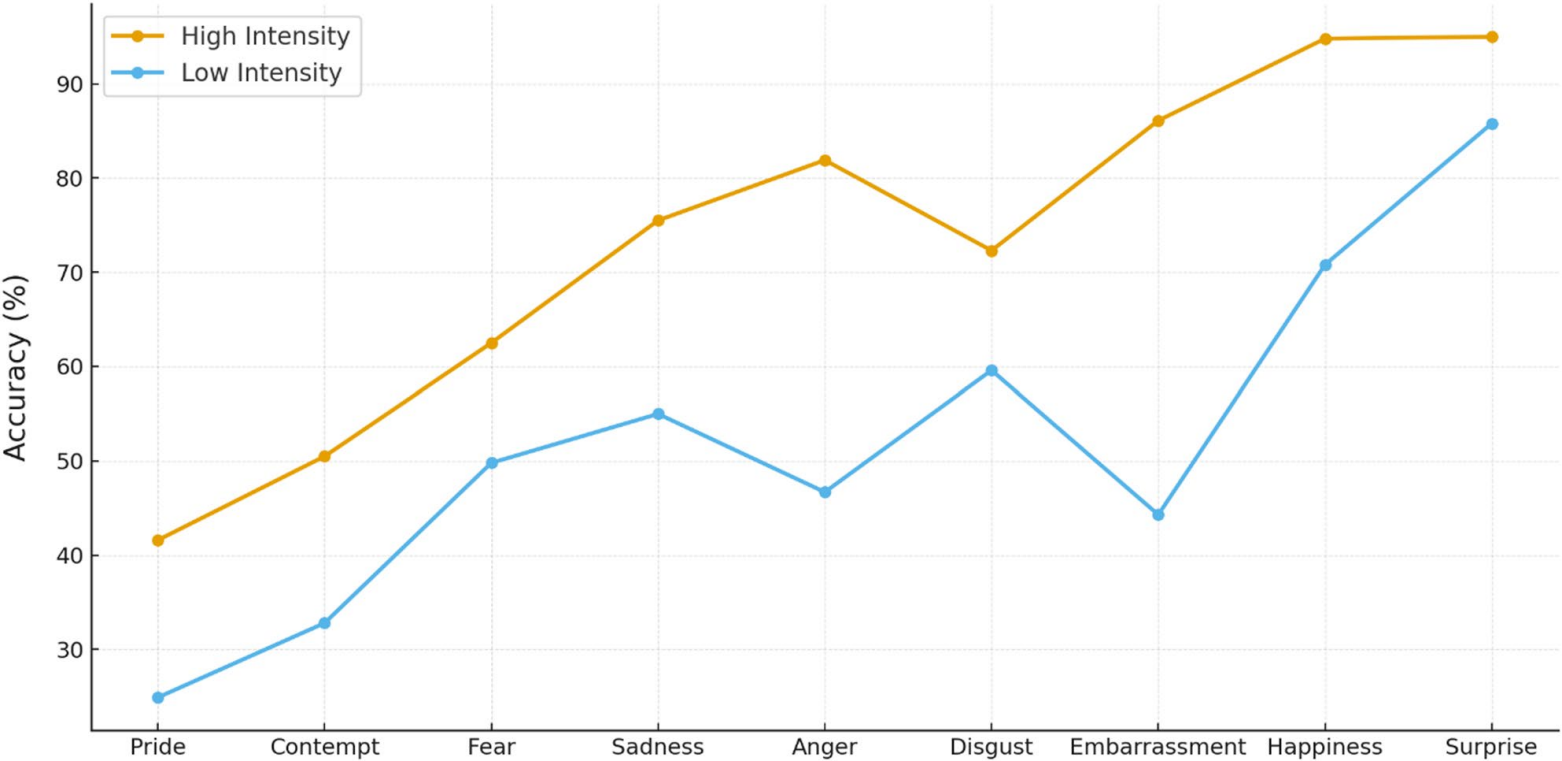
Emotion recognition accuracy (%) for high- and low-intensity dynamic facial expressions across emotions.

For high intensity, accuracy was highest for surprise (95.0%), followed by happiness (94.8%), embarrassment (86.1%), anger (81.9%), sadness (75.5%), disgust (72.3%), fear (62.5%), contempt (50.5%), and pride (41.6%). For low intensity, accuracy was highest for surprise (85.8%), then happiness (70.8%), disgust (59.6%), sadness (55.0%), fear (49.8%), anger (46.7%), embarrassment (44.3%), contempt (32.8%), and pride (24.9%) (see Fig. 1). Bonferroni-adjusted post hocs showed that, at high intensity, all pairwise contrasts were significant (all ps < 0.05) except disgust vs. sadness and happiness vs. surprise (both ps > 0.05). At low intensity, only anger vs. embarrassment and anger vs. fear were non-significant (both ps > 0.05).

#### FER and empathy

Pearson correlations between empathy subscales and FER are shown in Table 3 (Benjamini–Hochberg corrected across 44 comparisons). QCAE affective and cognitive empathy were not significantly associated with total FER or any specific emotion scores (all ps > 0.05). For the MET, total FER was not associated with affective empathy (*r* = −.04, *p* =.893) but was positively associated with cognitive empathy (*r* =.21, *p* =.042). At the emotion level, MET cognitive empathy showed weak positive correlations with embarrassment and pride (Table 3). Consistent with this, MET cognitive empathy remained positively associated with FER total accuracy when controlling for age and gender (partial *r* =.21, *p* =.002).

**Table 3:** Correlations between QCAE, MET and facial emotion recognition after correcting for multiple correlations

Scale	Variable	FER –Total		Emb	Sad	Pd	Con	Hap	Dis	Ang	Sur	Fr	Neu
QCAE	Affective Empathy		0.08	0.04	0.07	0.01	− 0.02	0.05	0.08	0.06	− 0.10	0.19	− 0.06
	Cognitive Empathy		− 0.08	− 0.11	0.06	0.07	0.01	0.13	0.00	− 0.03	− 0.16	0.08	− 0.06
MET	Affective Empathy		− 0.04	− 0.05	− 0.06	0.01	0.02	− 0.13	0.00	0.02	0.02	− 0.01	− 0.07
	Cognitive Empathy		0.21*	0.23**	0.08	0.21*	0.07	− 0.01	0.07	− 0.01	0.16	0.07	0.06

*FER* facial emotion recognition, *Emb* embarrassment, *Pd* pride, *Con* contempt, *Hap* happiness, *Dis* disgust, *Ang *anger, *Sur* surprise, *Fr* fear, *Neu* neutral. * *p* <.05. ** *p* <.01. *** *p* <.001

Stepwise regression assessed predictors of MET cognitive empathy. A model including total FER and age was significant, *F*(2, 224) = 31.18, *p* <.001, explaining 21% of the variance (R² = 0.21). Both overall FER (t = 3.33, *p* <.001) and age (t = 7.01, *p* <.001) were positive predictors. A second model including embarrassment, pride, and age explained 22% of the variance, *F*(3, 223) = 20.94, *p* <.001; embarrassment (t = 2.37, *p* <.05) and age (t = 6.52, *p* <.001) were significant positive predictors, whereas pride was not (t = 1.51, *p* =.132).

#### FER and psychopathic traits

Pearson correlations indicated no significant associations between PPTS-R (total or subscales) and FER accuracy (total or emotion specific) when examined separately for low- and high-intensity blocks (all ps > 0.05). Full results are provided in Appendix 1.

### Convergence between self-report and performance-based empathy

Within-measure empathy components were positively associated (QCAE, *r* =.42, *p* <.001), whereas MET affective and cognitive empathy were not significantly associated (*r* = −.06, *p* =.387). QCAE and MET affective empathy scales were positively correlated (*r* =.31, *p* <.001), their cognitive empathy scales were not significantly correlated (*r* = −.06, *p* =.356). Moreover, the affective empathy scale of MET was positively correlated with the cognitive empathy of QCAE (*r* =.25, *p* <.001).

## Discussion

The present study investigated the relationship between self-reported and performance-based measures of empathy and their associations with psychopathic traits and FER. The correlations and predictions involving empathy and psychopathic traits varied depending on the type of empathy measurement used. Both self-reported and performance-based measures of affective empathy were found to be negative predictors of psychopathic traits (but see note of caution regarding possible overlap of items between QCAE and PPTS-R). This finding aligns with previous studies highlighting deficiencies in affective empathy [15, 21, 59]. Although QCAE affective empathy correlated modestly with both affective and cognitive empathy on the MET, these associations are consistent with the view that affective and cognitive empathy are related yet distinct constructs [3, 4].

Cognitive empathy exhibited not only differences at the facet level but also overall disparities in its association with psychopathic traits when assessed using different methods. While self-report measures of cognitive empathy (QCAE) showed significant correlations with overall psychopathic traits, the opposite pattern emerged when examining overall performance-based (MET) results. Specifically, self-reported cognitive empathy was negatively associated with AR, CR and EGO (but see appendix 2 regarding potential overlap in items). The performance-based measure did not show any correlation with the subscales after correcting for multiple comparisons for both measurements. This might further explain the unclear relationship between cognitive empathy and psychopathic traits, as self-reported and performance-based assessed cognitive empathy did not correlate. Concerns are raised about the large number of studies that have employed self-report questionnaires to draw theoretical conclusions regarding the differing roles of affective and cognitive empathy [55].

When considered alongside prior work, the present pattern is broadly consistent with studies using the MET, which have typically reported reduced emotional empathy but little or no impairment in cognitive empathy in individuals higher in psychopathic traits [18, 30]. Likewise, findings from studies employing the QCAE across different psychopathy measures such as Triarchic Psychopathy Model (TriPM), generally show reliable reductions in affective empathy, with more variable associations for cognitive empathy [24, 35, 36].

However, while certain inconsistent findings can be attributed to the prevalent use of self-report measures for cognitive empathy, it’s also important to consider the characteristics of the sample and the psychopathy measures employed. In particular, the PPTS-R focuses on interpersonal and affective features of psychopathy and intentionally excludes overt antisocial or criminal behaviors. Although this approach avoids criterion contamination, it also means that the measure does not assess the disinhibition component, which contemporary models such as the Triarchic framework identify as a core psychopathy dimension - one that is strongly associated with antisocial behavior [34, 75]. Its omission in the PPTS-R means that our findings primarily reflect affective–interpersonal features rather than the full triarchic structure. Acknowledging this not only helps clarify the scope of the present results, but also highlights the value of examining differential associations across psychopathy dimensions in future research.

The present research also set out to investigate the relationship between empathy and psychopathic traits with FER. Before interpreting these associations, we verified that the FER task performed in line with well-established patterns in the literature. Recognition accuracy varied significantly across emotions and intensities, with socially complex emotions (e.g., contempt, pride) showing lower accuracy than basic emotions such as happiness and surprise [76, 77], supporting the reliability of the measure for subsequent analyses.

When examining associations between empathy measures and FER, only performance-based cognitive empathy (MET) showed a significant relationship with FER performance. Here, cognitive empathy was positively predicted by the ability to recognize facial expressions of embarrassment. The absence of a significant correlation between both self-reported and performance-based measures of affective empathy and FER suggests that the inconsistencies observed in prior research, reporting both positive [40] and zero correlation [78], may not be solely attributable to the type of measurement employed. Compared to other studies, these findings provide contributions to the literature as this study addresses some of the limitations observed in previous studies. For instance, despite the inclusion of a broader range of emotions and the utilization of dynamic facial expressions, which enhances ecological validity [57], the relationship between affective empathy and FER did not yield significant correlations.

Regarding cognitive empathy and FER, this study highlights the potential for divergent patterns based on the measurement type, underscoring the significance of employing performance-based tasks for empathy assessment. These findings suggest that previously reported associations between affective empathy and FER - particularly those relying solely on self-report measures - should be interpreted with caution, unless supported by additional evidence validating the self-report instruments in use. Moreover, while a correlation between empathy and embarrassment has been established in prior research [79], the present study extends this work by suggesting that recognition of embarrassment may be a particularly sensitive indicator of higher cognitive empathy. However, when the accuracy rate of participants on different emotions was investigated, embarrassment was not one of the emotions that were found to be harder to recognize, compared to emotions such as fear, contempt, or pride. Therefore, this relationship may not purely be attributed to its complexity. One potential explanation is that identifying embarrassment may demand heightened perspective-taking [80], a key aspect of cognitive empathy, especially in interpersonal contexts. Nevertheless, it is imperative to emphasize that further research is needed to validate this connection and to uncover the underlying mechanisms that elucidate the relationship between cognitive empathy and the recognition of facial signs of embarrassment.

The prediction regarding the relationship between FER and psychopathic traits remained uncertain due to conflicting findings in the existing literature [37, 48, 51, 81]. However, the present study has revealed no statistically significant association between psychopathic traits and the ability to recognize facial emotions, whether considering overall performance or the examination of individual emotions. These findings are consistent with other results obtained in this study, particularly the observation that FER is only correlated with cognitive empathy and that psychopathic traits do not demonstrate a relationship with general cognitive empathy score when assessed using performance-based tasks. While some previous research has reported impairments in recognising specific emotions such as sadness and fear among individuals with psychopathy, as posited by the Integrated Emotion Systems (IES) model [41, 82], our study aligns with others that have found no correlation between psychopathic traits and FER [50]. These results suggest that FER may not be the underlying factor contributing to affective empathy deficiencies within community samples characterised by moderate levels of psychopathic traits. Future research using longitudinal or clinical/high-trait samples is needed to examine this relationship more directly. It’s also important to consider that these findings might be attributed to the exclusion of antisocial or criminality traits from the psychopathy measure and the utilization of more ecologically valid FER tasks [83]. Additionally having a second scale that includes antisocial trait for comparison purposes, is something that can be investigated in future studies. Furthermore, in this study, the psychopathic traits scores ranged from 29 to 98 out of 112, with the mean of 58.62, the first quartile at 50 and the third quartile at 67. While this range reflects a heterogenous distribution of psychopathy scores, it’s significant to consider that scores, at the higher and lower ends of the scale could influence the study’s findings.

Contradictory findings once again emerged in the examination of the relationship between various empathy measurements, age, and gender. Age exhibited a negative correlation with self-reported empathy but demonstrated a positive association with performance-based measurements of empathy. Furthermore, females self-reported higher levels of empathy compared to males [84, 85], while no significant gender-based difference was observed in performance-based empathy measurements. These contradictory findings have introduced uncertainty regarding the relationship between age and empathy in the existing literature. Some studies have shown that affective empathy is not significantly linked to age [86], while others have reported an increase in empathy with advancing age [87]. Studies assessing cognitive empathy behaviorally have also yielded mixed results, including declines [88, 89], no differences [90], and even increases in empathy [91]. It is important to note that most of these studies encompassed participants up to the age of 80, with a few also involving children. Consequently, the findings of this study may not be directly comparable to those of previous studies, as the participants in this study fall within the age range of 18 to 60 years, with a slightly higher number of young adults compared to other age groups. Any observed age differences should be interpreted with this contextual difference in mind. Regarding gender differences in empathy, the findings of the present study are consistent with prior research utilizing self-report questionnaires, which typically demonstrate higher empathy scores among females [92]. However, this pattern does not consistently extend to studies employing physiological [93] or performance-based measures of empathy [85]. For instance, Michalska et al. [94] reported that although females scored higher than males on self-report measures of empathy, no significant gender differences were observed in neurophysiological indices of empathic arousal. These findings align with the current results, suggesting that gender differences in empathy are more pronounced when assessed through self-report measures, but tend to diminish or disappear when measured via performance-based tasks or neurophysiological methods.

Divergent results between self-report and performance-based empathy measurements might stem from limitations inherent in self-report scales, such as social desirability bias, limited self-insight, and the challenge of distinguishing affective empathy from cognitive empathy. It’s worth noting that gender differences in empathic reporting are predominantly observed in self-report measures, possibly influenced by societal gender role expectations, whether intentionally or unintentionally [95]. In addition to the tendency for females to report higher empathic levels and males to report lower levels, various individual and social factors may play significant roles in shaping these differences. When examining these results in the context of psychopathic traits, another layer of potential misinterpretation arises, given that the male participants exhibited significantly higher levels of psychopathic traits. Specific psychopathic traits, such as EGO and IPM, may further complicate self-awareness regarding empathic tendencies. For instance, a meta-analytical finding indicates there is no significant relationship between psychopathy and the inclination to self-report socially undesirable traits, commonly referred to as ‘faking good’ [96]. Furthermore, while the Questionnaire of Cognitive and Affective Empathy (QCAE) [14] has been proposed as an effective tool for distinguishing between affective and cognitive empathy, the findings suggest that self-reports may not consistently differentiate between these distinct components of empathy.

The current study extends the literature by providing a direct comparison of performance-based and self-report empathy within the same sample, and by examining these associations using a psychopathy measure that intentionally excludes antisocial or criminal criteria and ecologically valid FER task. This approach may help clarify why previous studies employing different psychopathy measures and empathy methodologies have produced divergent findings, particularly with respect to cognitive empathy.

### Clinical implications

Although the present study was conducted in a general-population sample, the findings may have indirect relevance for clinical research. Specifically, the consistent association between psychopathic traits and reduced affective empathy - across both self-report and performance-based measures - highlights the centrality of affective processes in psychopathic traits. In contrast, psychopathic traits were not related to FER accuracy in this community sample, indicating that FER ability appears preserved at moderate levels of psychopathic traits. Further work, including samples with a broader range of psychopathic traits, is needed to clarify when FER difficulties arise and how they relate to empathic functioning.

### Limitations and future directions

The current findings should be interpreted in light of several limitations. First, although individuals with a current or past diagnosis of anxiety, depression, or autism were excluded from participation, this screening was based solely on self-report. These particular conditions were selected a priori because autism spectrum conditions are associated with differences in both empathy and FER, whereas depression and anxiety are more consistently linked to difficulties in recognizing facial emotions [9, 63–65]. Their exclusion was therefore intended to minimize potential confounding influences on the socio-emotional measures used in the present study. However, this decision might limit ecological representativeness, as these conditions occur in the broader population. Moreover, the screening relied solely on self-report, and participants with undiagnosed conditions may still have been included. Future research using structured clinical interviews would allow more comprehensive screening across a wider range of conditions - including those not evaluated here, such as substance use disorders [97] - and would help determine the generalizability of the present findings to more heterogeneous samples.

Second, the assessment of psychopathic traits relied exclusively on self-report measures, which are susceptible to bias and may not fully capture the interpersonal and affective dimensions of psychopathy. Future studies should consider incorporating clinical or performance-based assessments that reflect core psychopathic traits - such IPM and EGO - without conflating them with antisocial or criminal behaviors [17]. Third, as all data were collected online, experimental control over environmental factors was limited and may have introduced additional variability.

The present study measured cognitive and affective empathy using both self-report measure (QCAE) [14] and the Multifaceted Empathy Test (MET), offering a multi-method approach that extends beyond reliance on a single assessment modality. While the MET provides a more objective evaluation of cognitive empathy through participants’ accuracy in recognizing emotions, its affective empathy component - despite being more ecologically valid than traditional questionnaires - still depends on participants’ self-reported emotional responses to static images. This limits its capacity to capture spontaneous or real-time empathic behavior.

A more fundamental limitation concerns the static nature of our experimental paradigms [98]. Both the MET’s assessment of affective empathy and our FER tasks relied on posed expressions presented in isolation, which fail to capture the dynamic, multimodal nature of real-world emotional communication. These measures conceptualize empathy and emotion recognition as individual, intrapersonal processes, overlooking their inherently interactive nature. This limitation is especially relevant in psychopathy research, where core social impairments may only become apparent during reciprocal, real-time interactions [23, 99]. This methodological limitation may help explain the divergent patterns of association observed across measurement approaches. Future studies should employ more ecologically valid paradigms that assess these constructs as dynamic, second-person processes [100]. Incorporating spontaneous emotional reactions during live interactions, dynamic video stimuli with synchronized vocal and facial expressions (e.g., infant cries) [101], or multimodal vocalizations (sighs, laughter) could better capture the complexity of real-world social cognition [83].

### Conclusion

The present study explored the correlation between psychopathic traits, and FER. Notably, these relationships exhibited considerable variability based on the specific measurement of empathy employed. Psychopathic traits displayed a negative correlation with both affective and cognitive empathy as assessed via self-report scales. However, it exhibited only a negative association with performance-based measurements of affective empathy. Interestingly, the connection between empathy and FER demonstrated significance solely for cognitive empathy when assessed through performance-based task, particularly in recognizing expressions of embarrassment. Notably, investigations into psychopathic traits and FER yielded non-significant results across both self-report and performance-based measure. In addition to the divergent findings between self-report and performance-based measures of empathy, it is worth emphasizing that these measures did not yield meaningful predictive interchangeability.

This study contributes significantly to the existing literature by illuminating the potential source of inconclusiveness within these relationships, specifically the exclusive reliance on self-reports of empathy. Furthermore, it underscores the importance of excluding antisocial or criminality traits when assessing psychopathy to attain more meaningful results. An important avenue for future research is the integration of diverse assessment methodologies, particularly those incorporating performance-based and socially embedded measures. Such approaches would move beyond an exclusive reliance on self-report instruments and provide a more ecologically valid account of how social interactions unfold in real time. Furthermore, replication and deeper investigation of the mechanisms underlying affective empathy deficits in psychopathy remain essential. Advancing this line of inquiry may not only clarify the etiology of these deficits but also inform the development of more targeted and effective intervention strategies. 

## Data Availability

All data utilized in this study are publicly available and can be accessed via the following GitHub repository: https://github.com/merve-utangac/Psychopathy_Empathy_ER.git.

## Appendix 1

**Table 4:** Pearson Correlations Between Psychopathic Personality Traits Scale – Revised (PPTS-R) Scores and Emotion-Specific Facial Emotion Recognition

FER Outcome	PPTS-*R* Total	Egocentricity	Affective Responsiveness	Interpersonal Manipulation	Cognitive Responsiveness
FER (total)	0.07	0.08	0.04	0.07	0.03
FER (high)	0.06	0.07	0.01	0.08	0.03
FER (low)	0.07	0.07	0.05	0.06	0.03
Embarrassment	0.06	0.09	0.03	0.07	−0.01
Sadness	−0.00	0.02	−0.01	0.02	−0.03
Pride	0.11	0.10	0.13	0.10	−0.00
Contempt	0.08	0.04	0.05	0.12	0.00
Happiness	0.03	−0.03	0.07	−0.04	0.12
Disgust	0.05	0.03	0.02	0.04	0.09
Anger	0.05	0.07	0.03	0.04	0.03
Surprise	−0.00	−0.01	0.02	−0.08	0.09
Fear	−0.10	−0.03	−0.16	−0.04	−0.08
Neutral	0.04	0.05	0.01	0.02	0.04

## Appendix 2. Confirmatory Factor Analysis (CFA)

A Confirmatory Factor Analysis (CFA) was conducted to further examine whether item overlap between the self-report measures (QCAE and PPTS-R) could account for the observed associations. The chi-square statistic indicated some degree of misfit, χ²(1246) = 2420, *p* <.001, which is not unexpected in larger samples. Approximate fit indices suggested a marginally acceptable model: RMSEA = 0.064 (90% CI [0.061, 0.068]), SRMR = 0.088, CFI = 0.770, and TLI = 0.755. Following conventional benchmarks, the RMSEA was within an acceptable range (< 0.08), whereas the CFI and TLI values were below recommended thresholds (> 0.90), indicating room for improvement. Inspection of factor loadings showed that most items loaded significantly on their respective latent constructs (all *p* <.001), with standardized estimates ranging from 0.30 to 0.81.

## Abbreviations

ADFES: Amsterdam Dynamic Facial Expression Set
AR: Affective Responsiveness
CE: Cognitive Empathy
CFA: Confirmatory Factor Analysis
CR: Cognitive Responsiveness
EGO: Egocentricity
FER: Facial Emotion Recognition
IPM: Interpersonal Manipulation
IRB: Institutional Review Board
MET: Multifaceted Empathy Test
PPTS-R: Psychopathic Personality Traits Scale–Revised
QCAE: Questionnaire of Cognitive and Affective Empathy
